# Association of serum levels of secreted frizzled-related protein 5 and Wnt member 5a with glomerular filtration rate in patients with type 2 diabetes mellitus and chronic renal disease: a cross-sectional study

**DOI:** 10.1590/1516-3180.2019.0304.R2.09122019

**Published:** 2020-06-01

**Authors:** Indira Rojo Báez, Daniel Omar Rivera Castro, Sarahí Salas Gutiérrez, Edgar Dehesa López, Adriana Aguilar Lemarroy, Luis Felipe Jave Suarez, Hipólito Castillo Ureta, Edith Hilario Torres Montoya, Vicente Olimón Andalón

**Affiliations:** I PhD. Professor, Department of Immunogenetics, Facultad de Biología, Universidad Autónoma de Sinaloa, Culiacán, Sinaloa, México.; II BSc. Biologist, Facultad de Biología, Universidad Autónoma de Sinaloa, Culiacán, Sinaloa, México.; III BSc. Biologist, Facultad de Biología, Universidad Autónoma de Sinaloa, Culiacán, Sinaloa, México.; IV MD. Research Investigator, Centro de Investigación y Docencia en Ciencias de la Salud, Culiacán, Sinaloa, México.; V PhD. Research Investigator, Centro de Investigación Biomédica de Occidente, Instituto Mexicano del Seguro Social, Culiacán, Sinaloa, México.; VI PhD. Research Investigator, Centro de Investigación Biomédica de Occidente, Instituto Mexicano del Seguro Social, Culiacán, Sinaloa, México.; VII PhD. Research Investigator, Facultad de Biología, Universidad Autónoma de Sinaloa, Culiacán, Sinaloa, México.; VIII PhD. Research Investigator, Facultad de Biología, Universidad Autónoma de Sinaloa, Culiacán, Sinaloa, México.; IX PhD. Research Investigator, Facultad de Biología, Universidad Autónoma de Sinaloa, Culiacán, Sinaloa, México.

**Keywords:** Sfrp5 protein, human [supplementary concept], Wnt5a protein, human [supplementary concept], Diabetes mellitus, type 2, Renal insufficiency, chronic, Diabetes mellitus, Anti-inflammatory cytokine, Diabetic nephropathy, ELISA quantification, Sfrp5, Wnt5a

## Abstract

**BACKGROUND:**

Diabetic nephropathy is a common complication of chronic kidney disease (CKD). ­Inflammation in the kidneys is crucial for promoting development and progression of this complication. Wnt member 5a (Wnt5a) and secreted frizzled-related protein 5 (Sfrp5) are proinflammatory proteins associated with insulin resistance and chronic low-grade adipose tissue inflammation.

**OBJECTIVE:**

To determine the correlation between serum Sfrp5 and Wnt5a concentrations and glomerular filtration rate in patients with type 2 diabetes mellitus and CKD.

**DESIGN AND SETTING:**

Cross-sectional, comparative and observational study in the Department of Endocrinology, Civil Hospital, Culiacán, Sinaloa, Mexico.

**METHODS:**

Eighty individuals with chronic kidney disease were recruited. Their serum Sfrp5 and Wnt5a concentrations were quantified using the enzyme-linked immunosorbent assay (ELISA) test. The statistical analysis consisted of the Mann-Whitney U test for independent samples and Spearman correlation, with statistical significance of P < 0.05.

**RESULTS:**

Serum Sfrp5 concentration continually increased through the stages of CKD progression, whereas serum Wnt5a concentration presented its highest levels in stage 3 CKD. Negative correlations between estimated glomerular filtration rate (eGFR) and serum concentrations of Sfrp5 (r = -0434, P = 0.001) and Wnt5a (r = -0481, P = 0.001) were found.

**CONCLUSIONS:**

There were negative correlations between serum Sfrp5 and Wnt5a concentrations and eGFR at each stage of CKD, with higher levels in female patients. This phenomenon suggests that Sfrp5 and Wnt5a might be involved in development and evolution towards end-stage renal disease.

## INTRODUCTION

Diabetes mellitus (DM) has come to be among the chronic degenerative diseases with the most significant increases in morbidity in Mexico over recent years.^1^ Within this, type 2 diabetes mellitus (DM2) is caused by a combination of insulin resistance and an inadequate compensatory insulin secretory response. DM2 is the most prevalent form of DM worldwide, and it leads to high rates of complications such as chronic kidney disease (CKD), along with mortality.^2^^,^^3^

Diabetic nephropathy is a relatively common complication of DM that, once established (as the diabetic nephropathy phase), is irreversible and progresses to CKD.^4^ In Mexico, the prevalences of both type 2 diabetes mellitus and diabetic nephropathy have increased over recent years. A previous study showed that the prevalence of diabetic nephropathy was 24% in an adult population among rural communities.^5^

Previous studies have shown that inflammation in the kidneys is crucial for promoting development and progression of diabetic nephropathy. During the physiopathological development of this condition, macrophages and T cells accumulate in the glomeruli and interstices, even in the early stages of the disease. This interstitial macrophage accumulation is strongly correlated with serum creatinine and inversely with renal function, thus creating a proinflammatory profibrotic stage and angiogenesis. Products that are secreted include tumor necrosis factor-α (TNF-α), interleukin (IL)-1 and IL-6, among others. Likewise, elaborate chemokines further direct migration of additional macrophages to the kidneys, thereby establishing an inflammatory cycle.^6^

Wnt member 5a (Wnt5a) is a proinflammatory protein belonging to the Wnt family. Presence of this protein has been correlated with macrophage release, insulin resistance and chronic low-grade inflammation within adipose tissue.^7^ On the other hand, secreted frizzled-related protein 5 (Sfrp5) is an anti-inflammatory protein that is mainly released by fat cells, which has also been linked with insulin resistance and chronic low-grade inflammation within adipose tissue, and which acts as an endogenous inhibitor of Wnt5a.^8^^,^^9^ There have been reports indicating that members of this family become altered in kidney diseases and in chronic inflammatory diseases such as fibrosis.^10^ However, no studies on the participation of Sfrp5 and Wnt5a in patients with chronic kidney disease have been conducted.

## OBJECTIVE

The aim of this study was to determine the correlation between serum Wnt5a and Sfrp5 concentrations and glomerular filtration rate in patients with type 2 diabetes mellitus and chronic kidney disease.

## METHODS

### Study population

A cross-sectional, comparative and observational study was conducted during the period from August 2014 to November 2015. This study was approved by the Ethics Committee of the Universidad Autónoma de Sinaloa, Mexico (date: October 20, 2014; approval number: 0161).

We included all the 80 patients who met the inclusion criteria of having previously been diagnosed with DM2 and CKD, in accordance with the Kidney Disease Improving Global Outcomes (KDIGO) criteria. These patients were recruited at the outpatient clinic of the Department of Endocrinology of the Civil Hospital of Culiacán, Sinaloa, Mexico.

The inclusion criteria were that the patients would be recruited during their regular check-up on their type 2 diabetes that had previously been diagnosed at the Department of Endocrinology of the Civil Hospital and needed also to have a diagnosis of chronic kidney disease in accordance with the KDIGO criteria. In addition, they needed to be 18 years of age or older and to have given voluntary written informed consent to their participation in this study. The exclusion criteria comprised situations in which participants had not given their written informed consent; absence of blood samples from the patient; and presentation of cancer, autoimmune diseases or infectious diseases.

### Collection of peripheral blood serum

Peripheral blood was obtained by means of venipuncture and was collected in sterile Vacutainer tubes without anticoagulant. These tubes were incubated for 30 minutes at room temperature. Subsequently, they were centrifuged at 2,500 rpm for 10 minutes. The soluble fraction was recovered and divided into aliquots of 0.5 ml, which were then stored at -80 °C until further use.

### Classification of the stages of chronic kidney disease

The glomerular filtration rate (GFR) was estimated using the Chronic Kidney Disease Epidemiology Collaboration (CKD-EPI) equation, with the variables of age, gender, race and serum creatinine. CKD was classified in accordance with the Kidney Disease Improving Global Outcomes (KDIGO) criteria, in terms of estimated glomerular filtration rate (eGFR), as follows: stage 1 (S1) = eGFR > 95 ml/min/1.73 m^2^; stage 2 (S2) = eGFR 60-89 ­ml/­min/1.73 m^2^; stage 3 (S3) = eGFR 30-59 ml/min/1.73 m^2^; stage 4 (S4) = eGFR 15-29 ­ml/­min/1.73 m^2^; and stage 5 = eGFR < 15 ml/min/1.73 m^2^.^11^

### Measurement of serum creatinine concentration

The serum samples were analyzed in a clinical chemical analyzer system (Ortho Clinical Vitros 250 Chemistry System, Minnesota, USA), using the creatinine dry reagent chemistry method. Around 500 µl of the serum sample was deposited in a microcuvette in this analyzer system. Previously, it had been calibrated and controlled for the creatinine analysis. The normal reference values were taken to be 0.5-1.5 mg/dl.^12^

### Measurement of serum Wnt5a and Sfrp5 concentrations

The serum Sfrp5 levels were determined using a commercial enzyme-linked immunosorbent assay (ELISA) kit (catalogue no. SEC842Hu; USCN Life Science Inc., USA) while the serum Wnt5a levels were determined using another commercial kit (catalogue no. E83549Hu; USCN Life Science Inc., USA). The volume of standard and serum used per well (undiluted) was 100 µl. Absorbance detection and quantification for the ELISA assays was done using a microplate reader (BioTek Synergy HT; BioTek Instruments, Inc., Winooski, USA) at a wavelength of 450 nm, in accordance with the manufacturer’s instructions.

### Statistical analysis

Descriptive statistical data analysis was used. The Shapiro-Wilk normality test was also used. Differences between pairs of groups were evaluated using the Mann-Whitney U test for independent samples, while comparisons among more than two groups were made using the Kruskal-Wallis test. Correlations between continuous variables were made using Spearman’s rank correlation coefficient. This descriptive statistical method was chosen because the data did not present normal distribution. Results were considered to be statistically significant when P < 0.05. The statistical analysis was performed using the Statistical Package for the Social Sciences (SPSS) software (version 22.0; NY, USA).

## RESULTS

The creatinine levels increased during the progression of the disease. The S1 kidney disease creatinine levels were significantly different from the S2, S3 and S4 creatinine levels. There were no significant differences between S2, S3 and S4. On the contrary, the glomerular filtration rate decreased during the progression of the disease. The rate in S2 was observed to be significantly different in relation to S3 and S4.

To determine the serum concentrations of Sfrp5 and Wnt5a in these patients with chronic kidney disease, they were grouped in each of the stages of the disease (Table 1).

**Table 1.** General parameters of patients according to staging of chronic kidney disease used in this study

Characteristics	Stage 1	**Stage 2**	Stage 3	Stage 4
Gender				
Female (n = 45)	8	17	14	6
Male (n = 35)	14	9	8	4
Age (years)				
25-39 (n = 5)	4	0	0	1
40-54 (n = 19)	7	7	3	2
55-69 (n = 39)	11	11	14	3
70-85 (n = 17)	0	8	5	4
Creatinine (umol/l)	0.71 ± 0.15^a,b,c^	1.02 ± 0.15^a^	1.33 ± 0.25	1.98 ± 1.40^c^
GFR (ml/min)	107.06 ± 8.6^b,c^	72.9 ± 7.9^e^	46.17 ± 16	25.06 ± 7.8^e^
Wnt5a (ng/ml)	0.19 ± 0.03^bc^	0.19 ± 0.02^d,e^	0.26 ± 0.08^b,d^	0.22 ± 0.01^c,e^
Sfrp5 (ng/ml)	11.60 ± 4.69^abc^	22.18 ± 9.94^a,e^	31.72 ± 32.60^a^	50.6 ± 223.5^ae^

GFR = glomerular filtration rate; Wnt5a = Wnt member 5a; Sfrp5 = secreted frizzled-related protein 5.

The data are expressed as the mean ± standard error of the mean (SEM). Results were statistically significant when P ≤ 0.05. ^a^stage 1 versus stage 2; ^b^stage 1 versus stage 3; ^c^stage 1 versus stage 4; ^d^stage 2 versus stage 3; ^e^stage 2 versus stage 4; ^f^stage 3 versus stage 4.

The Mann-Whitney U test was used to determine whether there were any significant differences in serum Sfrp5 concentrations between any of the CKD stages. There were significant differences between all of them except between stages 2 and 3. We also observed that the serum Sfrp5 concentration tended to increase with increasing stage (Table 1).

On the other hand, we observed that CKD stage 3 had the highest serum Wnt5a concentration. A multiple-comparisons test among the serum Wnt5a concentrations in relation to each CKD stage showed that there were significant differences between stages 1 and 3, stages 1 and 4, stages 2 and 3, and stages 3 and 4. The rest of the results did not show any statistically significant difference between the different stages of CKD secondary to DM2. However, it was observed that the Wnt5a concentrations were statistically significantly higher in the more advanced stages than in the earlier stages of chronic renal disease (stages 3 and 4 versus stages 1 and 2) (Table 1).

**Figure 1.**
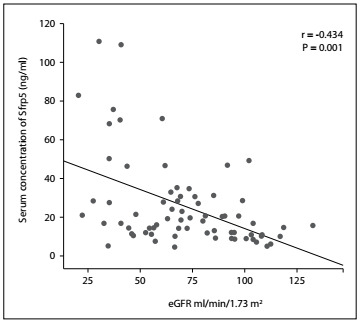
Correlation between serum concentrations of secreted frizzled-related protein 5 (Sfrp5) and estimated glomerular filtration rate (eGFR). Spearman’s rank correlation with P < 0.05 was used to determine statistical significance.

**Figure 2.**
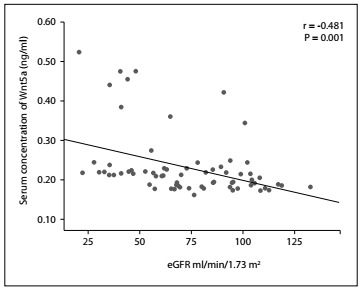
Correlation between serum concentration of Wnt member 5a (Wnt5a) and estimated glomerular filtration rate (eGFR). Spearman’s rank correlation with P < 0.05 was used to determine statistical significance.

The GFR of each individual was estimated and the serum Sfrp5 and Wnt5a levels were subsequently quantified to determine the correlation of these proteins with the clinical evolution of CKD patients with type 2 diabetes mellitus. Spearman’s rank correlation was used to identify the relationship between serum Wnt5a and Sfrp5 concentrations and glomerular filtration rate. From this, we found a statistically significant negative correlation between serum Sfrp5 concentration and eGFR (r = -0434, P = 0.001) (Figure 1) and between serum Wnt5a and eGFR (r = -0481, P = 0.001) (Figure 2) in these patients with chronic renal disease, i.e. eGFR decreased with increasing serum concentration of these proteins.

**Figure 3.**
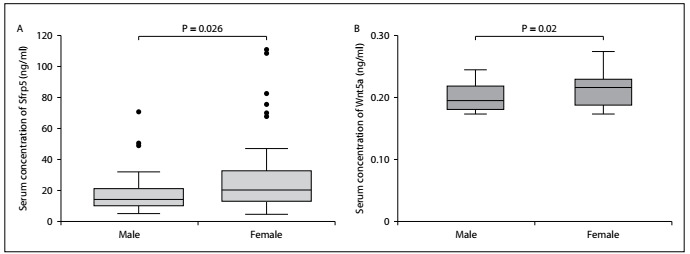
Serum concentrations of secreted frizzled-related protein 5 (Sfrp5) and Wnt member 5a (Wnt5a) according to gender among patients with chronic kidney disease. A) Serum Sfrp5 concentration; and B) serum Wnt5a concentration; differences between groups were ascertained using the Mann-Whitney U test and a P < 0.05 was used to determine statistical significance.

We also found that females had significantly higher serum concentrations of Sfrp5 and Wnt5a, in comparison with males (Figure 3).

**Figure 4.**
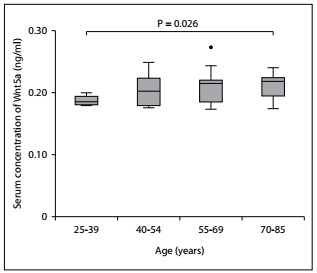
Serum Wnt5a concentration according to age ranges among patients with chronic kidney disease. Differences between groups were obtained using the Mann-Whitney U test and P < 0.05 was used to determine statistical significance.

We observed significant differences in serum Sfrp5 concentration, in relation to age, among these patients with chronic kidney disease. In contrast, significant differences in serum Wnt5a concentration were only found in relation to the age ranges of 25-39 and 70-85 years (P = 0.024). There were no statistical differences in serum Wnt5a concentration in relation to the age ranges of 40-54 and 55-69 years (Figure 4). Thus, we found that the serum levels of these proteins increased with the evolution of clinical symptoms in chronic kidney disease and with the age of the patients.

## DISCUSSION

The present study showed that the serum levels of Sfrp5 increased according to the stages of CKD. It is important to mention that our study was the first to find a correlation between serum Wnt5a levels and glomerular filtration rate in patients with type 2 diabetes mellitus.

Sfrp5 is an anti-inflammatory cytokine that is highly expressed in white adipose tissue.^13^ Its presence has been correlated with low-grade chronic inflammation in adipose tissue, obesity, insulin resistance, type 2 diabetes mellitus and cardiovascular diseases.^14^^,^^15^^,^^16^ In addition, presence of Sfrp5 has been correlated with chronic kidney disease, and we also demonstrated that the serum levels of SFRP5 were higher in patients with chronic kidney disease than in healthy patients.^10^

It has been reported that Wnt5a, which is a pro-inflammatory cytokine, promotes insulin resistance.^17^^,^^18^ Moreover, Wnt5a is secreted by macrophages, which are involved in the production of different pro-inflammatory cytokines and have been associated with chronic low-grade inflammation in adipose tissue and type 2 diabetes mellitus.^18^ In comparisons of serum Wnt5a concentration between healthy people and patients with type 2 diabetes mellitus, it has been reported that diabetic patients have higher serum Wnt5a concentration than that of control individuals.^19^

In this study, we observed that serum Wnt5a concentration was higher in the advanced stages of chronic kidney disease, particularly during stage 3. This behavior may have been due to an increase in renal damage and inflammation at this stage.^20^ Therefore, infiltration will have become more abundant at this stage, thus leading to release of increased production of different cytokines.^21^

During stage 4, the serum concentration of Wnt5a decreased, but not with any statistically significant difference. This result may have been due to the increased hypoglycemia of end-stage renal disease, which generates thickening of the basement membrane located in Bowman’s capsule.^22^ This makes the basement membrane more permeable to proteins and other macromolecules that are excreted in urine, which may explain why the serum concentration of Wnt5a is lower in stage 4 than in stage 3. The increases in the serum concentrations of SFRP5 and WNT5A were correlated with decreases in estimated glomerular filtration rate, i.e. there was a negative correlation between these serum concentrations and the glomerular filtration rate. Hence, we observed that increasing serum Sfrp5 concentration was correlated with progression of CKD. This coincided with what was reported in another study about a negative correlation between serum Sfrp5 levels and glomerular filtration rate in patients with chronic kidney disease.^10^

With regard to gender, the serum levels of Sfrp5 and Wnt5a were significantly higher in females than in males. This may have been because hormone levels are greater in females than males. In a study on an anti-inflammatory adipokine similar to Sfrp5, it was reported that there was a negative correlation between testosterone levels and adiponectin concentration in males.^23^ Importantly, most of the females included in our study were at or beyond the menopause. These stages give rise to greater inflammation in different cells of the immune system, such as macrophages, with release of pro-inflammatory cytokines like IL-6 and TNF-α.^24^ In addition, progesterone (a hormone involved in the menstrual cycle, pregnancy and lactation) induces expression of Wnt5a. Moreover, progesterone is the most commonly used hormone for treating menopausal symptoms.^25^^,^^26^

With regard to the correlation between the ages of these patients with chronic kidney disease and the serum concentration of Sfrp5, no significant differences were observed. However, there was a significant positive correlation with Wnt5a, given that with age progression the concentration of WNT5A also increased. In patients with type 2 diabetes mellitus and chronic kidney disease, their kidneys are the main organ affected because the main function of the kidneys is to filter waste and toxins out of the blood, which gives rise to persistent inflammation with increasing age.^27^

## CONCLUSION

This study showed that the glomerular filtration rate gradually decreased through the stages of progression of chronic kidney disease. It also demonstrated that there was a negative correlation between the serum concentrations of Sfrp5 and Wnt5a and the clinical stages of chronic kidney disease. In addition, Sfrp5 was seen to play an important role in the progression to end-stage kidney disease. However, there is a need to carry out complementary studies in order to demonstrate the participation of Sfrp5 and Wnt5a within the pathophysiology of chronic kidney disease.
